# Louisiana State Dental Society

**Published:** 1886-04

**Authors:** 


### LOUISIANA STATE DENTAL SOCIETY.

### ANNUAL MEETING FOR 1886.

REPORTED FOR THE INDEPENDENT PRACTITIONER, BY "MRS. M. W. J."

The first public meeting of the Louisiana State Dental Society was held in Tulane Hall, New Orleans. The meeting was called to order at 10 A. M., March 4th, and continued three days, with two daily sessions.

An address of welcome was delivered by Col. Wm. Preston Johnston, President of Tulane University. He bade the profession a hearty welcome to the precincts of the University, which was always ready to open wide its doors for anything in the line of educational progress. He said that education is the great civilizing and lifting power; that it has made dentistry what it is, an honored and honorable profession; one that requires the exercise of all the faculties of the mind in combination with the highest mechanical skill and ingenuity, and all for the alleviation of human suffering and the promotion of health; a profession to which all of the arts and sciences are tributary. He suggested, as a practical plan for the advancement of the dental profession in Louisiana, the institution in Tulane University of a one year's course, preparatory to the Dental College; a course which shall include English, Biology, Physiology, Physics, Mechanical Drawing and Manual Training, all to be taught by a corps of the ablest men drawn from the highest institutes of learning. The special advantages gained by the two latter branches would be the training of the fingers to that delicacy of touch so essential in their profession, and of the eye to accurate measurement and judgment of color and form; they would also be trained—not as mechanics, but as artists—in the working of both wood and metals, and in the plastic arts. If this suggestion met with favor, the privileges would be extended to both medical and dental students, and he hoped they would urge its adoption and accept its benefits.

Geo. J. Friedrichs, M. D., D. D. S., in his

PRESIDENTIAL ADDRESS, briefly reviewed the history of the society from its organization in 1878. He pointed out the advantages of association in consolidating the profession and advancing it in its great and humane work. He paid a graceful tribute to the Fathers of dentistry, whose names are inscribed upon imperishable tablets in the temple of fame. He pointed out the urgent necessity of securing effective legislation against the inroads of professional incompetents and unprincipled adventurers. He considered the detection and remedy of predisposing causes of dental caries a fit subject of public sanitation, and that it was the province of dental societies to shape public opinion in this direction. He was scathingly severe in his rebuke of

THE ADVERTISING DENTIST.

He said : "He is not a contented man, for he publishes to the world that a generous public has not awarded him his due share of employment; he is not an upright man, for he disregards the ethics of his profession; he is an unprincipled man, for he tries to raise his own status by debasing the standing of his fellow-practitioners; he is an arrogant man, in his presumption placing himself above being taught; he is a conceited man, for he judges others by his own standard; he is a selfish man, his whole soul wrapped up in self; he is a dishonest man, for he promises results which he knows cannot be attained, and thus he procures money under false pretenses.

The first paper read was the report of a case of Antral Catarrh, in the practice of Dr. L. C. Anderson, Lake Charles, La., which, after unsuccessful treatment for six or seven months with Iodoform, Boracic Acid, Carbolic Acid, etc., yielded to two weeks' daily treatment with Corrosive Sublimate, (2 gr. to the oz). This case had been under physician's treatment for three years. There had been no relapse during the year since the case was discharged as cured.

*Dr. J. S. Knapp*—Thought that comparatively few cases of antral diseases were permanently cured. Had met with some success in the use of wood creosote, three drops to a teaspoonful of alcohol in two oz. of water, injected daily. A diseased condition of the antrum was sometimes due to a dead molar, of which the roots had penetrated the floor of the antrum. In that case the tooth should be removed, and the antrum treated through the opening made by the roots.

*Dr. A. G. Friedrichs*—Considered drainage and cleanliness essential points. Failures are largely due to the neglect of patients in following directions.

*Dr. D. G. Parker*—Treated with a twenty per cent. solution of elixir vitriol. Described a case in which necrosed alveolar process followed typhoid fever. Three fingers could be passed through the opening into the antrum. Loose pieces of bone were removed for nine months. A perfect cure was finally effected, using only elixir vitriol.

*Dr. G. J. Friedrichs*—(from the chair)—Said that a congested condition due to common cold, which would get well without treatment, was often mistaken for antral trouble successfully treated.

Dr. Salomon read a paper entitled

PHYSICIAN AND DENTIST.

It was a discussion of the relationship between the two professions, and the difficulties of drawing the line and saying how far the province of one extends, and where the duties of the other begin. He cited numerous cases of mistaken diagnosis by the physician, of exostosis treated for a tumor, etc. In one case in his practice, a young girl, thirteen years old, had been under treatment for supposed mumps, having been confined to her bed for two weeks with the swelling, growing worse continually. Being sent for, he found the jaws so rigidly set that for two days it had been impossible to introduce the handle of a spoon between her teeth. Cold applications externally, with listerine as a mouth-wash, injected with a small syringe, and the administration of quinine, so relaxed the muscles that he was able, the next morning, to introduce the little finger. He found a large swelling extending from the first lower right bicuspid to the second molar. He applied a twenty-five per cent. solution of cocaine (Merck's) and was able, without much pain, to open the mouth with a Stellwagen separating forceps. He found two small temporary roots wedged between the second bicuspid and the molar. Extracting these, a copious discharge of pus followed, and the mumps disappeared in twenty-four hours.

The other case had been treated for months for a tumor underneath the ear, for which a surgical operation was advised. The upper right wisdom tooth was found to be slightly decayed, and its position somewhat abnormal. A fine, flexible, steel probe, following the direction of the roots, revealed a mass of soft deposit not unlike macerated bone. The extraction of the wisdom tooth was followed by the speedy disappearance of the tumor. A knowledge of oral surgery, on the part of the physician, or the moral courage to admit the lack of it, would have spared the patient much agony of mind.

He said that the men who make a trade of tooth-pulling, who parade in the newspapers as "The only man with the gas engine;" the "Hypnotic Air" quack; "The Champion Exposition Dentist," etc., have degraded the profession of dentistry, and made the medical profession justly afraid and ashamed to acknowledge it, even as a specialty; that a dentist to be justly entitled to the name, should not only know how to make beautiful fillings, but he should have a knowledge of the causes of caries, and should thoroughly understand general pathology and physiology; that without this knowledge he could not, for instance, diagnose the causes of the swelling of the gums in girl patients at a certain age, or under certain circumstances, and in his ignorance might scarify or even cauterize the gums; neither could he understand the dental troubles of pregnant or nursing women, or the marvelous changes in the saliva in such cases. He considered that the dental surgeon required the same degree of knowledge, intelligence, and judgment as the oculist or the aurist, and must have them if he would stand on a par with them in the medical profession. He agreed with Dr. Chapin A. Harris, that dentistry should be taught in medical colleges, with special chairs for oral surgery and operative and mechanical dentistry, as is now the case in the Universities of Michigan, Pennsylvania, and Maryland, and the Vanderbilt University, Tennessee.

He said that in this scientific age, to prove our claim to membership in a scientific profession, each individual should practice his profession on a scientific basis, from a scientific standpoint. On the other hand, he considered that the physician should have a more thorough knowledge of the effect of his medicines and treatment on the oral cavity and its contents, and especially of the relationship between the oral cavity and the uterus, and reflex action as seen in the pains in sound teeth during gestation. He should understand the effect of a continued hyperæmic condition of the blood-vessels upon tooth-structure; of diseased teeth upon the general health; of the foul air and gases generated by decayed teeth in causing tubercular conditions; the connection between diseased teeth and troubles of the eye or ear; he should be able to distinguish between nasal catarrh and alveolar abscess, etc.

Finally, he urged that dentists, individually, should strive for special improvement and higher education, reading the journals, working in associations, putting down quackery, etc. In that way only would dentistry receive recognition as a fruitful branch of the great tree of medicine.

Dr. Salomon brought before the society, as illustrating his position, a man who had fractured his jaw two years ago by lifting a chair with his teeth. Four months ago Dr. S. had examined his case, and advised the removal of all necrosed bone and the application of an interdental splint, with an outside plate. His advice was not taken, but two months later the man placed himself under the care of two eminent surgeons. During two months' treatment they had only removed the loose pieces of necrosed bone. The fracture is not reduced, and pus is still oozing freely. The case was examined by members of the society, and the present condition found to be as stated.

*Dr. A. G. Friedrichs*—Thought the physician not so much to be blamed, as proper instruction in dental specialties was not given in the medical course. Only the dentist who was also a medical graduate, could appreciate how little medical students were taught of dentistry, the structure of the teeth, and their use in mastication, as the initiative of digestion included it all. The physician may vainly treat for weeks, through incorrect diagnosis, cases that the dentist would comprehend and cure at once.

*Dr. J. G. McCulloch*—Thought it proved, by the cases cited, that the physician had far greater need of a dental education than the dentist of a medical course.

*Dr. D. G. Parker*—Thought that dentists could accomplish much in the right direction by calling the attention of physicians of their acquaintance to such cases.

*Dr. Jos. Bauer*—Thought that the dental colleges had culled from the medical curriculum everything essential to dental science. Anatomy, Physiology, Histology, etc., were thoroughly taught to dental students, and there is no necessity for a dentist to graduate as a physician. The degree of D. D. S. should rank as high as M. D. in their respective professions.

*Dr. G. J. Friedrichs*—Thought that the paper opened up a wide field in the ethics of both professions. That a thorough medical course was only the proper foundation or starting point of a dental education; that it would prove an invaluable help.

*Dr. O. Salomon*—Did not consider it necessary for the dental student to take the degree of M. D. Many Universities have dental departments, dental students taking the regular medical course in anatomy, physiology, etc., with special additional lectures in dental specialties, operative and mechanical.

Dr. Jas. S. Knapp read a paper entitled

CYLINDER FILLINGS.

He considered cylinders the best form of gold for arresting caries, accomplishing, by the wedging process, the most thorough exclusion of air and moisture, by the most expeditious method. The welding process is very slow, difficult of adaptation to the borders of the cavity, and involves an amount of pressure dangerous to frail walls, also necessitating the use of the rubber dam, which not more than ten men in the United States are capable of applying quickly and accurately in cases of more than ordinary difficulty, and which occasions great discomfort, and even pain to the patient, and much worry to the operator. He described in detail the method of making and using cylinders, having some of them cone-shaped, dovetailing them in insertion, and condensing by lateral pressure. He claimed that the duration of contour fillings of cohesive gold was very brief as compared with cylinder fillings. Contour fillings leave imperfect margins, causing the contiguous tooth substance to disintegrate. With cylinder fillings there is a saving in cost to the patient, of time and labor to the operator, and greater benefit to the tooth.

*Dr. O. Salomon*—Considers the use of the rubber dam a great advantage, especially the "depressed dam," even for cylinder fillings.

*Dr. A. G. Friedrichs*—Thinks one great advantage of cylinder fillings to be the rapidity with which gold can be inserted; that it is not essential to keep the cavity absolutely dry; that the gold being thoroughly impacted against the walls of the cavity, all moisture is driven out, and no matter how dry it might be kept during the operation, natural fluids oozing from the tubuli would create some moisture.

*Dr. J. R. Knapp*—Considered many points in the paper extremely extravagant. In the natural position of the teeth they touch each other only at one point, near the grinding surface or cutting edge. Gold, when replacing tooth-substance, must restore the natural form of the tooth, which cannot be done with cylinders. The latter method also necessitates cutting away tooth-substance to make space for operating. He said that the majority of patients prefer the rubber dam to wads of paper and rolls of napkins saturated with saliva. The labor bestowed upon contour fillings in giving a perfect finish is not for the purpose of display, but to give a hard, dense surface for wear, and which prevents particles of food from lodging where they would generate acids and produce new decay. Cohesive gold work is slow only because it is thorough and artistic. With the electro-magnetic mallet, gold can be impacted against frail walls that would not bear the pressure of cylinder filling. Teeth which are separated and sawed between would come together again, the teeth even moving in their sockets to effect contact, the whole physiognomy being thus changed for a lifetime by this malpractice of dentists in using a " cross-cut saw " between the teeth. We should leave the teeth as nature designed them.

*Dr. Jos. Bauer*—Though he used cohesive gold with the electric mallet, thought it might be well, in large cavities, to begin with cylinders and finish with cohesive gold. He did not consider it necessary to restore the contour of approximal surfaces of incisors and canines, leaving a V-shaped opening from the approximo-palatine surface. The teeth touching at the labial or buccal surface, there is no disfigurement, and they become self-cleansing. There is much of good in the cylinder method, and it would be more used if better known.

*Dr. Geo. J. Friedrichs*—Said that the reason why cylinders were not better known was because the method was not taught in our colleges. Gold could not be so condensed as to resist mastication; even eighteen karat gold will wear down in six months. He cited the case of a patient for whom he built down eleven teeth with cohesive gold, giving a masticating surface which wore down in six months so as to require rebuilding. Wearing down again in the same time, he made solid gold caps, with no better results. He then built down the cuspid only, opening the bite a quarter of an inch, and inserted artificial teeth, which have done good service for three years, and the cuspid building still remains good. The best of fillings are, he considered, only temporary, the majority not lasting more than ten years. He was opposed to restoration of contour, or "knuckling," where occlusion would drive it out of place or mastication wear it down.

*Dr. J. R. Knapp*—Said that if properly filled with cohesive gold, the gold and the tooth material would wear down evenly, with the margins intact.

*Dr. D. G. Parker*—Would obtain space by pressure with rubber wedges, and half-fill with cylinders, finishing with cohesive gold and contouring, using the rubber dam. In a frail bicuspid he would cut out the fissure between the cusps, building through with cohesive gold.

(TO BE CONTINUED.)

